# No association of GSTM1 null polymorphism with endometriosis in women from central and southern Iran 

**Published:** 2012-01

**Authors:** Seyed Morteza Seifati, Kazem Parivar, Abbas Aflatoonian, Razieh Dehghani Firouzabadi, Mohammad Hasan Sheikhha

**Affiliations:** 1 Department of Biology, Science and Research Branch, Islamic Azad University, Tehran, Iran.; 2 Research and Clinical Center for Infertility, Shahid Sadoughi University of Medical Sciences, Yazd, Iran.

**Keywords:** *Endometriosis*, *Glutathione-S-Transferase M1*, *Null genotype*

## Abstract

**Background:** Endometriosis is one of the most common gynecologic disorders. It is a complex trait and both genetic and environmental factors have been implicated in its pathogenesis. There is growing evidence indicating that exposure to environmental contaminants is a risk factor for endometriosis. Glutathione-S-Transferase M1 (GSTM1) is one of the genes involved in detoxification of endogenous and exogenous compounds.

**Objective:** Several studies have indicated an association between GSTM1 null mutation and endometriosis. In this study, the possible association between the GSTM1 gene null genotype and susceptibility to endometriosis in woman from central and southern Iran was investigated.

**Materials and Methods:** One hundred and one unrelated premenopausal women with endometriosis and 142 unrelated healthy premenopausal women without endometriosis were enrolled in the study. Genomic DNA was extracted from Peripheral blood in all subjects. GSTM1 null genotyping was performed by polymerase chain reaction (PCR).

**Results:** There was no significant difference between frequencies of GSTM1 null genotype in case and control groups (50.5% Vs. 52.1%, p=0.804). Furthermore, this genotype was not associated with severity of endometriosis in our sample (p=0.77).

**Conclusion** **:** further studies involving gene-environment and gene-gene interactions, particularly combination of GSTM1 and other GST gene family polymorphisms are needed.

## Introduction

Endometriosis (MIM 131200) is one of the most common gynecologic disorders and a cause of infertility in women. It is characterized by ectopic growth of endometrial tissue outside uterus cavity (1). The disease has been reported in 2-22% of women of reproductive age (2) and in 5-50% of infertile women (3). 

In spite of decades of clinical and basic research on endometriosis, little is known about its etiology and pathogenesis (4). The most widely held theory postulates that viable endometrial cells are carried to peritoneal cavity through Fallopian tubes by retrograde menstruation and are implanted there, giving rise to endometriosis (5). The fact that menstrual debris are found in peritoneal cavity of up to 90% of menstruating women casts some doubt on this theory (6). Endometriosis is a complex trait and both genetic and environmental factors have been implicated in this disease (7).

The role of genetic factors in endometriosis has been assessed and confirmed through numerous studies (8-13). These studies show a higher risk of endometriosis in first-degree relatives of endometriosis patients compared to general population (10, 11, 14). Twin studies provide further evidence of genetic propensity to endometriosis. These studies show a higher risk of endometriosis in monozygotic twins compared to dizygotic twins (15-17). Several studies have attempted to identify endometriosis susceptibility genes. Candidate genes studied thus far include genes involved in inflammation, steroid synthesis, and detoxification. Prominent among the latter are glutathione-S-transferases (GSTs) (7, 18). 

Numerous studies indicate a relation between environmental pollutants and endometriosis (19-21). It has been postulated that variants of genes involved in detoxification of these pollutants may play a role in endometriosis (22). GSTs are phase II detoxifying enzymes, catalyzing conjugation of electrophilic and hydrophobic compounds to reduced glutathione, thereby eliminating their toxicity (23). These enzymes are involved in detoxification of a wide range of toxic and carcinogenic substrates (24). 

Many of the known substrates of GSTs are xenobiotic substances and environmental pollutants. Different classes of these enzymes are specific for different substrates (25). Compounds like dioxins, which are ubiquitously present as known environmental pollutants throughout the world and impinge upon the food chain, are among GST's substrates (22, 26). In humans, cytosolic GSTs comprise six classes of enzymes including Mu (µ), Omega (ω), Pi (π), Theta (θ), and Zeta (ζ). Each of these classes is coded by one gene or gene subfamily (27). Glutathione-S-transferase M (GSTM) exists in 5 isoforms (GSTM1-5) and belongs to µ class. 

A gene cluster on chromosome 1 encodes these isoforms (28). GSTM1 gene is located on chromosome 1p13.3 and has four different alleles. GSTM1*A and GSTM1*B alleles are functionally similar and differ in only one amino acid (p.K172N). GSTM1*1×2 allele comprises two tandemly located genes, both of which are active (29). GSTM1*0 allele contains a deletional mutation. 

Due to the extent of this deletion, no mRNA or protein product is coded by the allele (30). A/A, A/O, B/B, and B/O genotype are all active and produce functional enzyme but O/O genotype causes a complete lack of enzymatic activity and is therefore called the null genotype (30, 31). 

Frequency of the null genotype differs in different ethnicities. It is estimated at 50% among Caucasians, 67% in Australians and 22% in Nigerians (32). GSTM1 enzyme metabolizes and detoxifies many exogenous and endogenous substrates, including dioxins, and also oxidative stress products produced during repair of ovarian epithelium after follicle rupture such as lipid hydro-peroxides, alkanes, and DNA hydro-peroxides (22, 26). Several studies have indicated an association between GSTM1 null mutation and endometriosis. 

GSTM1 null mutation has been associated with endometriosis in Russian, French, Indian, Chinese, Taiwanese, and Turkish women (33-40). No such association has been observed in English, Japanese, and Korean women (23, 26, 41, 42). To investigate the possible association between the GSTM1 gene null genotype and susceptibility to endometriosis and also its severity, we performed a case-control study in woman of central and southern Iran.

## Materials and methods

**Study population**

This case-control study was conducted between February 2009 and January 2011. On the whole, 101 unrelated premenopausal women with endometriosis were recruited at Yazd Mothers' Hospital and the Yazd Research and Clinical Center for Infertility, which are referral points for central and southern regions of Iran. 

Endometriosis was confirmed in subjects by laparoscopy after clinical examination. Laparoscopy was performed by an experienced gynecologist. Patients were staged (I-V) according to the guidelines of American Society for Reproductive Medicine (ASRM) classification system for endometriosis (1997). 

The control group consisted of 142 unrelated healthy premenopausal women undergoing Cesarean section or hysterectomy at the same centers with no history of endometriosis and without any lesion suggesting endometriosis during Cesarean section or hysterectomy. Age ranged from 20 to 43 years in endometriosis group and from 17 to 52 years in control group. 

The study was approved by the Ethics Committee and Research Committee of Yazd Research and Clinical Center for Infertility. Written informed consent was obtained from all participants.

**DNA analysis**

Peripheral blood from patients and controls was collected in EDTA-containing tubes. Genomic DNA extraction was performed using AccuPrep^®^ Genomic DNA Extraction Kit (Bioneer, South Korea) based on manufacturer's manual. GSTM1 genotyping for gene deletion was carried out by PCR. Part of the GSTM1 gene inside deletion area was amplified using primers 5'-GAACTCCCTGAAAAGCTAAAGC-3' (forward) and 5'- CTTGGGCTCAAATATACGGTGG-3' (reverse) (Cinnagen, Iran(. 

To check for the success of amplification, a set of β-globin gene primers [5'-G A A G A G C C A A G G A C A G G T A C - 3' (forward) and 5'-C C A C T T C A T C C A C G T T C A C C - 3 ' (reverse)] was added to each PCR tube which amplified a 268 base pair segment of this gene. Each amplification reaction contained 100ng genomic DNA, 1× PCR buffer [50 mM KCl, 10 mM tris-HCl (pH 8.4)], 1.5mM MgCl2 ، 200 μM dNTP, 10 pmol of each primers, and 1.5 U Taq DNA polymerase (Cinnagen, Iran( in a final volume of 25 μl. 

Amplification was performed with initial denaturation at 94ºC for 5 min, followed by 35 cycles of amplification which was performed at 94ºC for 1 min, 60ºC for 1 min and 72ºC for 1 min and a final extension at 72ºC for 10 min, using Mastercycler (Eppendorf, Germany). PCR products were detected by 1.5% (w/v) agarose gel electrophoresis. 

In homozygote non-null and heterozygote subjects a 219 base pair band is observed in agarose gel. The absence of this band (in the presence of β-globin PCR product) indicates the homozygote null genotype.

**Statistical analysis**

Fisher's exact test was used to compare genotype distributions between endometriosis and controls. Two-sample t-test was performed to compare age, Body Mass Index (BMI), age at menarche, parity and infertility between the endometriosis and control groups. 

P-value<0.05 was considered statistically significant. All analyses were conducted using the Statistical Package for Social Science version 11.5 (SPSS Inc., Chicago, IL).

## Results

Of the total of 101 patients, 15 (14.8%) had stage I (minimal), 39 (38.6%) had stage II (mild), 24 (23.8%) had stage III (moderate) and 23 (22.8%) had stage IV (severe) endometriosis. 76 patients (75%) suffered from dysmenorrhea, 28 (27.7%) from dysparonhea and 9 (8.9%) from pelvic pain. In 25 (24.8%) patients neither of these symptoms was observed. 

No statistically significant difference was observed in the age and infertility of patient and control groups but there were significant differences in BMI, age at menarche, and parity between the two groups (Table I). GSTM1 null genotyping was carried out successfully for all patients and controls. In table 2, frequency of null genotype in GSTM1 gene has been shown for 101 endometriosis patients and 142 controls. There was no statistically significant difference in the frequency of GSTM1 null genotype between patient and control groups (p=0.804). The frequency of GSTM1 null genotype was 45% in patients with advanced stages of the diseases (stages III and IV) and 55% in patients with the early stages (stages I and II). This difference was not statistically significant (p=0.77).

**Table I:** General characteristics of endometriosis patients and control group

**Characteristic**	**endometriosis**	**control**	**p value**
Age (yr) (mean±SD)	28.95 ± 5.115	29.82 ± 8.716	0.372^a^
BMI (kg/m^2^) (mean±SD)	24.09 ± 3.725	26.18 ± 4.788	0.001 a
Age at menarche(yr) (mean±SD)	13.48 ± 1.299	13.84 ± 0.919	0.019 a
Parity (n) (mean±SD)	0.356 ± 0.769	2.458 ± 1.447	<0.05 a
Primary infertility [n (%)]	70 (76.9)	7 (63.6)	0.457 b
Secondary infertility [n (%)]	21 (23.1)	4 (36.4)	

a t test

b Fisher's exact test

**Table 2:** Frequencies of GSTM1 null and non-null genotypes in endometriosis patients and control group

**GSTM1 genotype**	**Endometriosis**					**Controls**	**p-value** a
	**Stage I**	**Stage II**	**Stage III**	**Stage VI**	**Total**		
Null (%)	6 (40)	22 (56)	13 (54)	10 (43)	51 (50.5)	74 (52.1)	0.804
Non-null (%)	9 (60)	17 (44)	11 (46)	13 (57)	50 (49.5)	68 (47.9)	

a Fisher's exact test

**Figure 1:**
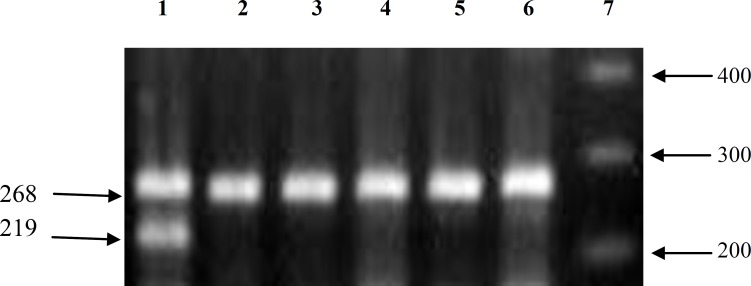
Agarose gel electrophoresis of the products of PCR. The 219 bp band shows presence of GSTM1 gene (lane 1). The 268 bp band is a fragment of β-globin gene, which gives a positive PCR control. Lane 7 is 100bp DNA size marker

## Discussion

The role of genetic factors in endometriosis has been assessed and confirmed through numerous familial and twin studies in different populations (10, 11, 14-17) and several studies have implicated GSTM1 gene as a possible candidate gene for susceptibility to endometriosis (18). 

We performed a case-control study in woman of central and southern Iran to test the hypothesis that the GSTM1 gene null genotype is associated with susceptibility to endometriosis or severity of it. Our study showed no significant difference between the frequencies of GSTM1 null genotype in case and control groups (50.5% versus 52.1%, p=0.804). Also, this genotype was not associated with severity of endometriosis in our sample (p=0.77). This is in accordance with the OXEGENE Collaborative study in England (2001) (26), and studies performed by Baxter *et al* (2001) in Southern England (41), Morizane *et al* (2004) in Japanese women (23), and Hur *et al* (2005) in Korean women (42). These studies found no association between GSTM1 null genotype and endometriosis. On the other hand, Baranov *et al* (1996) and Ivashchenko *et al* (2003) reported such an association among Russian women (33, 43). 

Subsequent studies by Baranova *et al* (1999) in French women (44), Lin *et al* (2003) and Peng *et al* (2003) in Chinese women (37, 38), Hsieh *et al* (2004) in Taiwanese women (39), Babu *et al* (2005) and Roya *et al* (2009) in Indian women (35, 36), and Aban *et al* in Turkish women (40) yielded similar results. Recently, a study performed in Rasht in northern Iran also showed such an association (45).These different results in different populations are to be expected. Association between a gene variant and a disease might only be manifest in populations with a particular genetic and environment background. 

Circumstances like linkage disequilibrium, gene-gene interactions, and gene-environment interactions can lead to failure of replication of results in association studies in populations with different genetic and environment backgrounds (46). There is growing evidence indicating that interaction of xenobiotic compounds present in different environments with variants of genes involved in their detoxification may change the risk of endometriosis (47). For example, Roya *et al* (2009) studied polychlorinated biphenyls (PCBs) and GSTM1 polymorphism and their possible role in the pathogenesis of endometriosis in Indian women. They found that women with GSTM1 null phenotype and higher concentrations of PCBs might have an increased susceptibility to endometriosis (36).

Taking these considerations into account, further studies in a variety of populations, involving gene-environment and gene-gene interactions, particularly combination of polymorphisms of GSTM1 and other types of GST gene family are needed to elucidate the relation of GSTM1 polymorphisms and endometriosis. 
